# Editorial: Neurodevelopment and preterm birth

**DOI:** 10.3389/fneur.2024.1412711

**Published:** 2024-04-24

**Authors:** Giulia Spoto, Antonio Gennaro Nicotera, Ambra Butera, Gabriella Di Rosa

**Affiliations:** ^1^Unit of Child Neurology and Psychiatry, Department of Biomedical Sciences, Dental Sciences, and Morphofunctional Imaging, University of Messina, Messina, Italy; ^2^Unit of Child Neurology and Psychiatry, Department of Maternal-Infantile, University of Messina, Messina, Italy; ^3^Unit of Child Neurology and Psychiatry, Department of Chemical, Biological, Pharmaceutical, and Environmental Science, University of Messina, Messina, Italy

**Keywords:** prematurity, neurodevelopment, white matter injury, gray matter, preterm

According to the World Health Organization, in 2020, the global rate of preterm birth ranged from 4% to 16%, and its complications represented the leading cause of death among children under 5 years of age (1). Despite the advances in neonatal care, children born preterm show a considerably higher risk of adverse outcomes compared to term-born babies. Notably, the risk of mortality and morbidity increased according to the degree of prematurity (2).

Premature birth can result in short- and long-term consequences, including poor health and growth, respiratory and gastrointestinal complications, and neurodevelopmental impairments that persist into adulthood. Particularly, motor, cognitive, communicative, and emotional/behavioral skills can be compromised, causing a significant burden on these children, their caregivers, and the healthcare systems (3).

Even though robust research has been conducted to address the underlying causes of prematurity, little change has been observed in the global preterm birth rate during the last decade (2). Numerous clinical and environmental factors have been considered as etiological aspects of prematurity (4). Perinatal factors such as brain injury, lower birth weight, late-onset sepsis, necrotizing enterocolitis, patent ductus arteriosus, and bronchopulmonary dysplasia have all been associated with adverse neurodevelopmental outcomes. In addition, exposure to the neonatal intensive care unit (NICU) environment can impact the neurodevelopmental outcome since children are exposed to reduced positive sensory stimuli (i.e., nurturing contact with the parents), and increased negative sensory exposures (bright and noisy environment, painful procedures). In very preterm babies, longer hospitalization in the NICU has been linked with worse cognitive, language, and motor outcomes at 1–2 years of corrected age (5).

In the last few decades, a growing body of research has focused on early detection tools to achieve prompt and accurate diagnosis and provide a timely intervention, optimizing neuroplasticity and function. These include neuroimaging, Prechtls' General Movements Assessment (GMA), and Hammersmith Infant Neurological Examination (HINE), which are currently considered the most validated assessments to diagnose cerebral palsy, the most severe motor outcome of prematurity (6).

Magnetic resonance imaging (MRI) is particularly effective in detecting nervous system lesions. Commonly, hypoxic-ischemic lesions occurring in preterm infants result in brain injuries involving both white and gray matter (7). Cystic periventricular leukomalacia and intraventricular hemorrhage (IVH) have been historically described as the most frequent preterm white matter injury, especially in children born very preterm or very low birth-weighted (8). Nowadays, more subtle lesions have been reported, such as diffuse microscopic punctate lesions, decreased white matter volume, and thinning of the white matter tracts (9). The pathological mechanism underlying the white matter damage of prematurity is the disturbed maturation of oligodendrocytes, especially of the oligodendrocytes progenitor cells (OPCs) (10). This connectivity damage can also alter the gray matter structures, including the cerebral and cerebellar cortex, thalamus, hippocampus, and basal ganglia (9, 11). Advanced techniques, such as functional MRI and diffusion-weighted imaging (DWI), have been recently used to understand the neurodevelopmental alterations underlying the long-term sequelae of prematurity (12). In this Research Topic, Zhao et al. used the diffusion tensor imaging (DTI), DWI, and diffusion kurtosis imaging (DKI) to assess the white matter development in 26 preterm infants and 26 full-term infants. Interestingly, they found that DKI is more useful in diagnosing delayed white matter development in premature infants than traditional techniques. Parameters such as the mean kurtosis (MK) and radial kurtosis (RK) allowed a more comprehensive characterization of the microstructural changes, especially in the posterior limbs of the internal capsule (PLIC) and anterior limbs of the internal capsule, proving to be superior to fractional anisotropy in diagnosing premature infants with delayed brain development. MK and RK were also more sensitive in analyzing the gray matter, playing an important role in detecting subtle structural changes of thalamic neurons in premature infants. Alternatively, Zhang et al., in their study on 15 preterm infants with low-grade IVH vs. 35 premature infants without intracranial hemorrhages, found that the synthetic MRI is also very sensitive in detecting microstructural modifications in the white matter of premature children with IVH. In their study, the analysis of T1 and T2 relaxation times and proton density values have been found excellent in detecting alterations in the PLIC, central white matter, and cerebellum. These findings support the notion that synthetic MRI might provide early prognostic biomarkers for neurodevelopmental disorders in preterm children.

In parallel to neuroimaging, clinical assessments and blood biomarkers have been studied in premature infants in order to early detect neurodevelopmental trajectories. GMA and HINE are emerging as efficacious detection tools in populations of preterm babies with low-risk of cerebral palsy, yet at risk of different neurodevelopmental disorders (13, 14). In the paper of Dicanio et al., significant correlations were found between the GMA at 3-months corrected age and the quality of movements, posture, and tone assessed with the HINE at 3-, 6-, and 9-months corrected age evaluations. Furthermore, the HINE showed that precocious visual and auditory abilities assessed with the Cranial Nerves subsection predicted communicative skills and auditory function, influencing the early language development in 1 year old preterm babies.

Regarding the blood biomarkers, Dong et al. focused their attention on serum peptide expression in the neonatal umbilical cord of children with hypoxic-ischemic encephalopathy (HIE). Their study identified potential endogenous functional peptides that may be involved in the progression of HIE and could be used as potential biomarkers. Among them, 21 peptides belong to the fibrinogen family, which is known to inhibit the differentiation of OPCs and, thus, myelination.

In the matter of myelination, Wang et al. conducted a remarkable study proving that transplanted human OPCs restored the myelin of injured white matter and led to neurobehavioral improvements in a mouse model of premature white matter injury. These results provide a new and promising perspective for the treatment of premature children.

Prematurity is a critical issue, given its long-term sequelae that profoundly impact patients' lives, families, and healthcare systems. This highlights the constant necessity for innovative interventions to enhance premature children's quality of life.

## Author contributions

GS: Data curation, Writing – original draft, Writing – review & editing. AN: Supervision, Writing – review & editing. AB: Data curation, Writing – review & editing. GD: Conceptualization, Supervision, Writing – review & editing.
